# Barriers and Facilitators to Vaccine Equity Amidst the COVID-19 Vaccine Rollout in the United States

**DOI:** 10.3390/ijerph21121588

**Published:** 2024-11-28

**Authors:** Rachael Piltch-Loeb, Josefina Nuñez Sahr, LaRon E. Nelson, David Vlahov, Robyn R. Gershon

**Affiliations:** 1 Institute for Implementation Science in Population Health (ISPH), City University of New York (CUNY), New York City, NY 10027, USA; 2 Department of Environmental, Occupational, Geospatial Health Sciences, City University of New York (CUNY), New York City, NY 10027, USA; 3 Yale School of Nursing, New Haven, CT 06477, USA; laron.nelson@yale.edu; 4 Justice, Community Capacity & Equity (JuCCE) Core, Center for Interdisciplinary Research on AIDS, Yale School of Public Health, New Haven, CT 06510, USA; 5 Department of Epidemiology, New York University School of Global Public Health, New York City, NY 10003, USA; rg184@nyu.edu

**Keywords:** COVID-19, vaccine equity, community engagement, public health, healthcare access, qualitative study

## Abstract

State and local health departments were responsible for ensuring equitable distribution of the COVID-19 vaccine. This qualitative study aimed to identify the challenges, strategies, disappointments, and successes in achieving equity for hard-to-reach and at-risk populations. Using a purposive sampling strategy, 16 individuals affiliated with health departments across nine states, each holding leadership roles in vaccine distribution, were interviewed between late 2021 and mid-2022. The key factors promoting vaccine equity included (1) inviting community members to serve on vaccine advisory groups to participate in decision-making; (2) utilizing pre-existing community relationships and spaces to facilitate the planning and distribution of the vaccine; and (3) establishing and building upon community outreach to support accessibility and uptake of the vaccine. The barriers included (1) a lack of clarity on vaccine prioritization criteria; (2) language/communication access; and (3) the initial focus on mass vaccination sites for vaccine delivery. The stakeholders also highlighted potential facilitators for increasing equity in future vaccine rollouts. Overall, community engagement emerged as a critical factor in ensuring equity during disaster response efforts.

## 1. Introduction

In January 2021, amidst the global standstill caused by the COVID-19 pandemic and the initial stages of the vaccine rollout, the United States Centers for Disease Control and Prevention (CDC) declared their commitment to vaccine equity, defining it as “ensuring fair and just access to COVID-19 vaccination” [1]. This brought into focus the need for strategies to address barriers to vaccination, especially among racial and ethnic minority members who may have been less likely to access or accept the vaccine due to a myriad of factors including racism and discrimination, lack of healthcare access, systemic mistrust in the government and medical system, and transportation or logistical barriers. The government’s commitment to equitable distribution was especially important in light of early findings suggesting that Black or African American and Hispanic or Latino Americans exhibited a lower likelihood of of receiving the vaccine than White Americans, despite experiencing higher rates of severe COVID-19 morbidity and mortality [2].

In the United States (U.S.), to help increase COVID-19 vaccine access, national organizations provided guidance to health departments [3] on vaccine outreach strategies to reach marginalized populations. The CDC provided information on specific ongoing programs [4] and the Federal Emergency Management Agency (FEMA) shared materials on setting up accessible mass vaccination sites [5].

Each jurisdiction took direction from national objectives and priorities while tailoring responses to local contexts, resulting in a variety of approaches to vaccine rollout at the state, local, and tribal levels. The challenge was formidable; localities had to manage vaccine distribution and follow federal guidelines, while navigating uncertainty in supply, storage, delivery, staffing, and funding for the vaccine, all the while confronting questions on vaccine safety, vaccine efficacy, and improving vaccine uptake [6].

Despite national and local efforts, disparities in vaccine uptake were rampant across sociodemographic groups and continue to this day [7]. The purpose of this study was to identify the facilitators and barriers to equitable vaccine distribution from key stakeholders engaged in leadership roles to identify strategies that might improve equity in vaccine access and uptake for jurisdictions confronted with launching future mass vaccination campaigns.

## 2. Materials and Methods

To start, we contacted, via an updated email list, the Directors of the Department of Health or Vaccine Initiatives in all U.S. states. The email invited them to participate in this study directly or to delegate a qualified individual from their department. The response rate was very low, and our sampling strategy shifted to utilizing a modified snowball technique. Using this new strategy, we were able to recruit an initial 5 individuals, who in turn led to the final recruitment of 16 individuals, some from the same state but located in different regions and cities of the state.

The semi-structured interview guide addressed three general topics: (1) strategies that were implemented to increase vaccination rates; (2) specific outreach activities to increase vaccine access in vulnerable populations; and (3) logistical problems that were of the greatest concern. This study was approved by the NYU Institutional Review Board and was conducted in late 2021 to mid-year 2022.

Based on the initial three topic areas, which were drawn from the CDC Guide for Community Partners [4], a resource guide for community organizations engaged with COVID-19 vaccination programs in racial and ethnic minority communities, we identified 10 interview questions included in the semi-structured script. The interviews, which generally lasted about 45 min, were digitally recorded, professionally transcribed verbatim, and then checked for accuracy. Our analysis plan was based on Grounded Theory [8] and employed the “constant comparative” method to identify themes that emerged from the interviews [9]. First, one researcher read and manually coded the first five transcripts to refine the codes. Next, using an inductive approach, two coders then independently coded each transcript, using constant comparison to further refine and expand upon the themes and to incorporate emerging insights. Any discrepancies in coding were discussed and resolved by members of the team. We used this iterative coding process to examine each item on the interview guide and then synthesized the results into two broad categories: facilitators and barriers to vaccine equity.

## 3. Results

A total of sixteen individuals from nine different states (reflected in Table 1), holding leading positions in vaccine distribution within their jurisdictions, underwent semi-structured Zoom interviews.

From their accounts, we identified three main factors promoting vaccine equity, three factors acting as barriers, and several future considerations. These are presented in Table 2 and explained below.

### 3.1. Factors That Promoted Vaccine Equity

Three consistent factors were identified as promoting vaccine equity, all based on strengthening and relying on community relationships.

#### 3.1.1. Developing Vaccine Advisory Groups

Vaccine advisory groups that were able to launch early and effectively were those that built upon the health department’s pre-existing networks. Their effectiveness and speed were facilitated by existing trust with community members and leaders.

*“We incorporated community engagement from the very beginning. Before any vaccine doses were even delivered to Oregon, we were planning sessions with communities, with specific groups, specific racial and ethnic groups as well as individuals with disabilities.”*

Vaccine Advisory Groups also included broad representation and multiple viewpoints, enabling them to develop a strategy they considered inclusive and ethical, as seen in the following quote:

*“[The vaccine task force] was charged with talking about the ethics and the process that would be used for prioritizing groups to receive the vaccine… It represented a broad swath or segment of society. So, there were people from the major health organizations. There were individuals representing community groups, community stakeholder groups. So native Hawaiian, Pacific Islanders, Filipino groups, the underrepresented homeless. I mean it was just a very large group of people. There were lawyers, people from the state agencies. You know, the Attorney General’s office…. that group then met, and they did a lot of work, and they came up with, well, who would get the vaccine first?”*

In some cases, this meant establishing a dedicated space where existing community relationships could formalize around a shared mission:

*“We knew there were all these other avenues and seats at the table like Medical Association, other state agencies, groups to give input into planning, but there was not a community place for input. So, we created the Vaccine Implementation Collaborative. It was truly a place to get feedback and input into decisions from community. In the beginning we had 400 people attending meetings”*

#### 3.1.2. Utilizing Pre-Existing Community Relationships and Spaces to Facilitate Planning and Distribution of the Vaccine

Vaccine advisory groups or task forces were set up to help prioritize vaccine distribution and, in some cases, these advisory groups assisted in the actual vaccine rollout and helped to identify facilities and locations that local community members would find most convenient.

*“We called it VOTE: The Vaccine Operations Team Equity. And they were specifically working with community-based organizations and faith-based organizations to set up these vaccination events that the community partner hosts. It’s not like, OHA [Oregon Health Authority] is doing this thing, come to it! It’s, my neighborhood center is doing this thing or the church is doing this thing and you can come get vaccinated. And it’s sponsored by an organization that the community knows, it’s vouched for by community leaders. And so that’s been a really important way in working with communities of color as well as the disability community in getting folks connected with vaccines.”*

#### 3.1.3. Establishing and Building upon Community Outreach Mechanisms to Support Accessibility of the Vaccine, Including Advertising Availability in Local Languages

Community engagement efforts, especially those built on pre-existing community relationships, were a critical part of vaccine planning and distribution, though community engagement may incorporate many different strategies and approaches. These efforts included setting up vaccination stations in local community spaces and meeting people where they were, as illustrated by the following quote:

*“….we set up vaccination stations and mobile sites at various churches or community centers throughout Dekalb and Fulton Counties. Again, to try to increase the accessibility to testing sites in these under-resourced communities. For example, like we set up at churches and they got a lot of buy-in from the pastors or leaders in that church community. So, I think having that community representative or that community leader that the rest of the community trusts, I think that was really helpful.”*

This commitment to community engagement was also reflected in efforts to reach residents in their preferred languages:

*“And so, I think what the county is doing well is we are advertising on buses, in newspapers, in different languages. San Mateo County is just south of San Francisco, and so we have a diverse community in that there are people who speak Tagalog, Mandarin, Cantonese, Spanish. I think the county has done a great job of getting the message out to various communities.”*

In some cases, local outreach included door-to-door campaigns to reach people who might not be embedded in the community or who might have disabilities or other functional and access needs that prohibit their participation in routine vaccination sites. This was also a good approach for discussing vaccine hesitancy issues.

*“…I wanted to mention a neighborhood-based outreach effort. It is modeled after political campaigns’ ground games. So basically, there are canvassers who are going door-to-door in high priority ZIP Codes talking to people about vaccines and having conversations and trying to get folks to kind of follow through with getting vaccinated and connecting them to local resources. So, I think this program has now been going for six weeks and they’ve knocked on 33,000 doors and had 11,000 conversations. And they’re getting really incredible feedback about the reasons why people haven’t gotten vaccinated yet.”*

### 3.2. Barriers to Vaccine Equity

Three factors that were barriers to vaccine equity were noted as follows: (1) vaccine prioritization criteria; (2) language/communication access; and (3) the focus on mass vaccination sites for vaccine delivery.

#### 3.2.1. Lack of Clarity on the Vaccine Prioritization Criteria

The lack of clarity on the criteria for initial vaccine distribution to priority groups was problematic. Because vaccine supply initially fell far short of demand, the Centers for Disease Control provided guidance for distribution [8]. The guidance prioritized healthcare workers, residents of long-term care facilities, those over 75 years of age, and front-line workers. However, this centralized prioritization often failed to align with individuals’ lived experiences, leading to additional confusion:

*“Folks didn’t feel like they were represented in the criteria. So, the language being used to describe someone with a disability who was eligible for phase 1A vaccine wasn’t how someone with a disability [might] identify. So, it felt like solving a riddle to figure out if you were eligible for the phase 1A vaccine.”*

Moreover, some materials contained conflicting information, with different sources offering incomplete or contradictory guidance:

*“I would say that the [prioritization] infographic wasn’t great because it resorted to that shorthand and it left people out. And so even if someone pulled up the 10-pager and said, ‘no, I’m pretty sure that I’m in phase 1A group 3, subcategory D because I received in-home services for my disability. Like I’m included in this’. And then the vaccinators would say, ‘no, but you’re not in this infographic’. I want to give people the benefit of the doubt and say that everyone was doing the best they could with the information that they thought was available. Infographics are good for communicating with the public, they are bad for communicating with providers. Providers need to not do the shorthand.”*

Finally, inconsistencies in distribution led to confusion and frustration within communities. As the following quote illustrates, some vaccine sites operated outside of these guidelines, enabling low-risk individuals to access vaccines intended for more vulnerable populations:

*“I remember when the rollout first happened, it was not within the healthcare system, but I remember there were certain schools, where they were administering the vaccine. And I remember seeing people posting on Facebook groups going, ‘XYZ high school… is giving out vaccines.’ And I remember thinking at that time, wait a second, we have a shortage. How are people publicly posting this? They were like ‘you can go in, it’s a walk-in’. And I was like, wait, that makes no sense. And then I saw folks who were healthy, not elderly, they were just walking in getting this. And I was like, who’s running this site? Like that’s not how it’s supposed to be going. So, it’s like even though we specified it should be going to a certain subpopulation, it wasn’t getting to them.”*

#### 3.2.2. Language Communication Access

Some individuals who required language accommodations were not able to be helped. Even though the importance of providing language accessibility was noted beforehand, assuring access was a persistent challenge.

*“I think a different communication access needs to be implemented. I think diversity needs to truly be cherished. And I mean diversity in communication. Whether a person prefers working with an interpreter. Spanish people need interpreters. Deaf/blind individuals need interpreters. Just like hearing people or deaf people, providing an option to be able to communicate via text to know when perhaps an appointment for a vaccine is available. Or to communicate via email. Adding those additional options would increase the methods to be able to access that information.”*

Even when translators or interpreters were available, other logistical constraints could create a barrier for their work to be effective. As one participant described, the high demand for services and privacy concerns made it challenging to provide adequate translation support:

*“And you can only call the translator so many times. Especially when you have a line of 200 patients standing outside the hospital. It’s difficult to get the translator online. And then you have to think about privacy and things. So, you can’t even put it on the speaker on the phone and have them translate because, for privacy purposes, it’s not like an enclosed area. And since health information is being disclosed you have to be very careful. So yes, the language barrier, that was huge.”*

#### 3.2.3. Limitations to Mass Vaccination Sites

Mass vaccination sites can increase efficiency by situating all of the vaccine services in one location. However, these sites presented challenges, especially for people with transportation barriers and access and functional needs. These challenges were compounded by logistical issues in reaching remote areas or people with limited mobility, as can be seen in the following quote:

*“…In the beginning when there was a limited supply, there was a lot of discussion about people in very remote areas. Now, on Maui is the Road to Hana. It would take you three hours to drive on this one-way road and if another car comes you have to pull your car over to the side. How are those people going to come down? But then how do you get vaccine up there?”*

Physical and digital access appeared to be a problem in urban areas as well. By relying heavily on vehicle access and digital tools, the system overlooked individuals who faced transportation and technology barriers, exacerbating disparities in vaccine access:

*“Here, Dodgers Stadium was used as a vaccine administering site. And that was cool. You had masses of people, but then it was sometimes like a wait of an hour and some people don’t have that one hour to actually give up and go all the way over there or don’t have a car. And some were like only vehicle-administering hubs. So …we saw that disparity in that sense and we weren’t getting folks vaccinated who didn’t work certain jobs, didn’t have access to a vehicle, were dependent on public transportation, who didn’t have access to a computer and therefore it wasn’t convenient for them to just scan a QR code.”*

Similarly, logistical constraints and strict rules at mass vaccination sites did not account for the special needs of people with physical disabilities or those requiring special accommodations. While mass vaccination sites were designed to streamline the process for large groups, they often lacked the flexibility needed to accommodate the complex realities of individuals, as exemplified by the following quotes:

*“I’ve heard of several accounts of situations with a drive-through, but there were strict rules. We saw that a lot with the mass vaccine sites that were staffed with DOD [Department of Defense] and FEMA [Federal Emergency Management Agency], you know really stripped top-down rules about not entering the vehicle. But if it was a van of a nursing facility and folks… You know, you have to take a step up into it. But it’s interesting because it wasn’t always a no.”*

*“Perhaps they were able to go get their first dose at a mass vaccine site because they borrowed someone else’s van. But then, since they borrowed someone else’s van, that made them ineligible for the second dose to be in-home.”*

### 3.3. Future Considerations

Throughout this study, the respondents also shared their perspectives on how to improve vaccine equity in the future. Their answers focused on three areas: (1) the need for strategies that ensure access to *all* members of the community, (2) recognizing the role of nurses and their potential impact on community members who are vaccine-hesitant and facilitating mass vaccinations at mass vaccine sites, (3) ensuring access for large numbers of people while still addressing the needs of vulnerable populations, such as the elderly and those with disabilities, and (4) stressing the importance of jurisdictions to make an institutional commitment to equity by appointing equity officers to the vaccination campaigns.

1. Strategies that ensure access to all members of the community have been published [10,11]. State and local plans should incorporate these and other strategies to address populations that have traditionally been overlooked in mass vaccination campaigns.
2. Recognizing the role of nurses.

Professionals across jurisdictions brought up nursing as a critical profession to improve vaccine access and distribution. Pullis et al. [12] underscores the important role nurses can play in the community in terms of addressing vaccine hesitancy and stresses the need for nurse training to build strong communication skills to do so effectively. There have also been recent calls for nurses to receive training in disaster management in general [13] and to help supervise and manage mass vaccine sites [14]. As one participant (an experienced nurse) noted:

*“I said, you know, you don’t really need all that many doctors. When you get a lot of patients you need nurses. And I don’t hear anybody saying we need nurses.”*

3. Ensuring access for large numbers of people while still addressing the needs of the most vulnerable members of the community.

*“…. locating sites that are closer [to transportation], [for] older adults, people with disabilities, transportation to site is a barrier. Making sure that our sites are fully ADA [Americans with Disabilities Act] compliant or having plain language and accessible signage, easy to navigate, comfortable for people of all abilities. There are strategies to make sure that restrooms and drinking water are all accessible. But I think one of the things that we did early and throughout were using mobile vaccination teams for individuals who are homebound or otherwise unable to easily travel to a health clinic, pharmacy or one of our vaccination sites.”*

4. Jurisdictions should explicitly commit to equity.

*“And so, … greatest good for the greatest number…. And that is just so contrary to these principles of equity…when you start being strategic and being committed to addressing and supporting communities that are most disproportionately impacted, thinking about what these barriers are, that’s harder. And it takes more time to be intentional and to be committed. And that’s necessary. But that’s really tough to explain and to commit to in an emergency where everything is on fire.”*

*“…. I’ll say that in terms of flipping the switch and turning into a response mode, one of the strategies we have is that we have an Equity Officer. So, for those who are familiar with the incident command structure, you have your public information officer and your safety officer and all these different roles that you would use in any kind of a hazard. Whether it be a pandemic or a winter storm or a wildfire or earthquake or whatever. We have embedded, over the years, and I think we were one of the first jurisdictions in the country to prominently place an Equity Officer at the top in a very leadership role.”*

## 4. Discussion

This study engaged with sixteen key stakeholders across nine U.S. states to identify the facilitators and barriers to equitable vaccine distribution during the initial COVID-19 vaccine rollout. The major finding involved highlighting partnerships with local community organizations and identifying convenient locations to access vaccination as pivotal to the success of their vaccine rollouts. This aligns with the existing literature that emphasizes the importance of building trust and engaging with communities by recognizing local sociocultural dynamics and leveraging community-based organizations. In a qualitative study with community health workers from seven different U.S. states, Mayfield et al. discussed the positive impact community health workers can have with respect to ensuring equity, underscoring their role as trusted, accessible liaisons within communities that contribute grassroots information critical to addressing health disparities and supporting policies [15]. Using convenient locations to site vaccine outreach contrasts with ad hoc, mass vaccination sites that might not be readily or comfortably accessible.

Despite many successes in rolling out the vaccine under difficult circumstances, many barriers to equitable vaccine distribution were identified, particularly affecting people with disabilities and non-English speakers. Language and accessibility barriers at vaccination sites were a source of inequities. Many sites lacked accessible features for people with access or functional needs. Mass vaccination sites, which were prioritized in the initial phase of the vaccine rollout, inherently did not have an equity focus because they were geared towards reaching the masses rather than focused on the specific needs of vulnerable populations. This was complicated by guidance from the federal government on vaccine eligibility that was seen as confusing and not always adaptable to meet the needs of people with physical or mental disabilities. Vaccine shortages coupled with confusing, changing, and competing priority groups led to dissension. Occasionally, implementing local prioritization strategies was challenging. This confusion was exacerbated when media information (especially infographics) on the priority groups was inconsistent. Guidelines on vaccine eligibility that were also advertised and promoted in different formats, such as full reports, pamphlets, or infographics, proved to be a source of confusion.

These findings align with a 2021 field experiment conducted during the vaccine rollout, in which audit testers across U.S. states assessed accessibility and communication at COVID-19 vaccination sites. The results showed that frontline staff in state health departments and mass vaccination sites often provided inconsistent information about vaccination requirements and procedures, with additional barriers encountered by Spanish speakers because of a lack of translation and/or negative attitude from staff [16].

To enhance equity in future vaccine distribution efforts, several strategies were recommended. A statewide equity strategic plan could ensure that equity considerations are integrated from the outset. Appointing an Equity Officer within the Incident Command System (ICS) structure could also provide dedicated oversight and coordination of equity-focused initiatives.

The participants also emphasized the important role of community health workers, including nurses, in mass vaccination programs. From helping supervise outreach to advising on and supervising mass vaccination sites, nurses could play an outsized role in these campaigns. They should not be limited to simply administering vaccines. Ensuring their communications and disaster response training so that they can help plan and respond to these types of events should be a national priority.

Overall, one of the most important lessons learned from local and national efforts to achieve vaccine equity is the necessity of a multifaceted approach that engages health systems, public health, and community systems, from both a strategic and an implementation standpoint. With the strategies discussed here, future vaccine rollouts can become more equitable.

## 5. Conclusions

This study highlights the critical role of partnerships with local community organizations and the use of convenient, accessible vaccination locations in achieving vaccine equity. These findings align with the existing literature emphasizing community engagement and trust-building, particularly through leveraging community-based organizations. While many successes were noted in vaccine distribution, significant barriers—especially related to language accessibility and the needs of people with disabilities—persisted. Mass vaccination sites, prioritized early on, often failed to address the specific needs of vulnerable populations, compounded by unclear federal guidance on vaccine eligibility. To improve equity in future vaccine rollouts, the participants recommended the development of statewide equity plans, the appointment of Equity Officers, and a greater role for community health workers, particularly nurses, in overseeing outreach and operational logistics. This study underscores that a comprehensive, multifaceted approach involving both public health and community systems is essential for achieving true equity in vaccine distribution.

## 6. Limitations

This study has several limitations. First, we interviewed stakeholders from only nine U.S. states, which may limit the generalizability of our findings to regions not represented in our sample. Additionally, seven out of the nine states included are politically left-leaning (“blue states”), potentially influencing both the perspectives shared by the participants and the public health strategies discussed. This political skew may affect the applicability of our findings in more conservative regions. Furthermore, these results are specific to the U.S., which has a unique healthcare system lacking a centralized primary health network that relies heavily on pharmacies, universities, and other private institutions to support the COVID-19 vaccine rollout. While these findings may offer insights for countries with similar decentralized health systems, caution is needed in extrapolating them to countries with different healthcare infrastructures.

## Figures and Tables

**Table 1:** Number of interviewees by U.S. state.

State	# of Interviewees
California	4
Connecticut	1
New York	2
Washington	2
Hawaii	1
Florida	1
Georgia	2
Massachusetts	1
Oregon	2

**Table 2:** Barriers, facilitators, and future considerations from the thematic analysis.

Factors that Promoted Vaccine Equity	
1	Developing vaccine advisory groups
2	Utilizing pre-existing community relationships and spaces to facilitate planning and distribution of the vaccine
3	Establishing and building upon community outreach mechanisms to support accessibility of the vaccine, including advertising availability in local languages
Barriers to Vaccine Equity	
1	Lack of clarity on the vaccine prioritization criteria
2	Language communication access
3	Limitations to mass vaccination sites
Future Considerations	
1	Elaborating strategies that ensure access to all members of the community
2	Recognizing the role of nurses
3	Ensuring access for large numbers of people while still addressing the needs of the most vulnerable members of the community
4	Jurisdictions should explicitly commit to equity

## Data Availability

The raw data supporting the conclusions of this article will be made available by the authors upon request.
